# Movement Disorders in Brain Sagging Syndrome Due To Spontaneous Intracranial Hypotension: A Review

**DOI:** 10.5334/tohm.914

**Published:** 2024-09-06

**Authors:** Abhigyan Datta, Alfonso Fasano, Abhishek Lenka

**Affiliations:** 1 Department of Neurology, University of Minnesota, Minneapolis, MN, US; 2 Edmond J. Safra Program in Parkinson’s Disease, Morton and Gloria Shulman Movement Disorders Clinic, Toronto Western Hospital, UHN, Toronto, Ontario, CA; 3 Division of Neurology, University of Toronto, Toronto, Ontario, CA; 4 Krembil Brain Institute, Toronto, Ontario, CA; 5 Department of Neurology, University of Texas Southwestern Medical Center, Dallas, TX, US

**Keywords:** Brain sagging, Intracranial hypotension, Movement disorders, Ataxia, Parkinsonism

## Abstract

**Background:**

Spontaneous intracranial hypotension (SIH), a treatable condition that stems from spinal leakage of cerebrospinal fluid, usually presents with orthostatic headache, nausea, vomiting, dizziness, and tinnitus. A subset of patients, especially those with sagging of brain structures (“brain sagging syndrome”), develop several movement abnormalities. As SIH is treatable with epidural blood patch (EBP), movement disorders neurologists should be familiar with this syndrome.

**Method:**

The authors performed a literature search in PubMed in July 2024 using the Boolean phrase- *((“Brain sagging”)OR(“Intracranial hypotension”))AND((((((((((“Movement disorders”)OR(“Involuntary movements”))OR(“Tremor”))OR(“Dystonia”))OR(“Chorea”))OR(“Ballismus”))OR(“Myorhythmia”))OR (“Tic”))OR(“Ataxia”))OR(“Parkinsonism”))*.

**Result:**

We tabulated 21 case reports/series that highlighted the presence of movement disorders. The most reported phenomenology is gait unsteadiness. While it usually emerges in the background of the classic SIH symptoms, rarely, patients may present with isolated gait dysfunction. Tremor is the second most reported phenomenology with postural and kinetic tremor being the common subtypes. Holmes tremor has also been reported in SIH. Other reported phenomenologies are parkinsonism, chorea, and dystonia. One study reported a unique phenomenology i.e. compulsive repetitive flexion and breath holding in 35.3% of the patients. In majority of the patients, EBP resulted in substantial clinical and radiological improvement.

**Discussion:**

Brain sagging syndrome due to SIH may present with a wide range of movement disorders. Mechanical distortion of the posterior fossa and subcortical structures result in the emergence of such movement abnormality. SIH adds to the list of conditions that result in “treatable movement disorders.” Therefore, movement disorders neurologists should be versed with the diagnosis and clinical features of this condition.

## Introduction

Spontaneous intracranial hypotension (SIH) is a well-described clinical and radiological syndrome that usually stems from the spinal leakage of cerebrospinal fluid (CSF) [1,2,3]. Very rarely, SIH may occur due to cranial leakage of CSF [4]. While orthostatic headache is the most common symptom of SIH, patients may present with a plethora of other symptoms attributed to low CSF pressure, such as nausea/vomiting, neck pain/stiffness, hearing problem, tinnitus, dizziness, vertigo, diplopia, and back pain [2,3,5]. The decrease in CSF volume may result in ventricular collapse and brain sagging, occasionally leading to serious consequences such as distortion of the diencephalon and brainstem. This can lead to a constellation of radiographic findings suggestive of brain sagging, which, in addition to the classic symptoms of SIH, may result in behavioral and cognitive changes similar to those in behavioral variant frontotemporal dementia (in which case it is referred to as frontotemporal brain sagging syndrome or FBSS), and can rarely lead to stupor and coma in severe cases [6,7]. There is some ambiguity in nomenclature, as subsequent papers have referred to FBSS simply as brain sagging syndrome (BSS), however, BSS defined by radiographic criteria may not always present with classic symptoms of FBSS. Thus, in this article, we use the term BSS in the context of SIH with classic radiologic features (described below), regardless of whether they exhibit the clinical phenotype of FBSS. As the clinical profile of SIH/BSS often partially overlaps with several other neurologic conditions, misdiagnosis of this condition is very common [8].

In approximately 10% of the patients with SIH, movement disorders of various phenomenologies may emerge [9]. Both hypokinetic (parkinsonism) and hyperkinetic (tremor, chorea, stereotypies such as repetitive neck flexion) movement disorders and ataxia have been reported in patients with SIH, and these symptoms can occur concurrently or in the absence of symptoms of FBSS. These movement disorders are thought to stem from the mechanical distortion and stress on relevant deep brain structures and the cerebellum. While BSS is often refractory to conservative measures like hydration and caffeine intake, there is considerable improvement when treated with lumbar epidural blood patch (EBP), as well as targeted blood patches for patients with identified CSF leak sites [2,10,11]. Consequently, movement disorders associated with BSS are treatable, underscoring the importance for movement disorders neurologists to be familiar with the key features of BSS.

This article aims to comprehensively review the spectrum of movement disorders associated with BSS, shedding light on the clinical presentations, underlying mechanisms, and therapeutic approaches in managing this complex condition.

## Method of Literature Search

The authors performed a comprehensive literature search in PubMed in July 2024 using the Boolean phrase- ((“Brain sagging”) OR (“Intracranial hypotension”)) AND ((((((((((“Movement disorders”) OR (“Involuntary movements”)) OR (“Tremor”)) OR (“Dystonia”)) OR (“Chorea”)) OR (“Ballismus”)) OR (“Myorhythmia”)) OR (“Tic”)) OR (“Ataxia”)) OR (“Parkinsonism”)), which yielded 37 articles. Additional articles were identified by scrutinizing the references of all included articles. After excluding 17 articles that were unrelated to the theme of this review, we shortlisted 26 articles that reported involuntary movements of various phenomenologies in the context of BSS. Additional search in EMBASE database using the keywords “Brain sagging syndrome” and “Intracranial Hypotension” did not yield additional articles suitable for the review. Case reports and case series in which individual patient data were described in detail, were tabulated as per specific movement disorders phenomenologies (21 studies, Tables 1, 2, 3, 4).

**Table 1:** Summary of publications on SIH/BSS that have reported gait dysfunction as a predominant symptom.


AUTHOR, YEAR	AGE/SEX	TYPICAL SIH SYMPTOMS NOTED?	TYPICAL FBSS SYMPTOMS NOTED?	OTHER SYMPTOMS	TYPE OF GAIT ABNORMALITY	KEY EXAMINATION FINDING	IMAGING ABNORMALITIES	TREATMENT	IMPROVEMENT OF SIH/FBSS SYMPTOMS?	IMPROVEMENT OF MOVEMENT DISORDERS?	FOLLOW UP BRAIN IMAGING

Nowak et al. 2003	50/M	No	no	Visual blurring	Paroxysmal episodes of severe unsteadiness when standing and walking	Not mentioned apart from moderate unsteadiness	B/L subdural hygromas. CSF leak at T12–L1levels	EBP	–	Complete resolution	Not done

Peng et al. 2004	50/F	Yes (orthostatic headache, nausea, vomiting)	Yes (short term memory loss)	Sensorineural deafness	Gait ataxia	Impaired tandem gait, MMSE 26/30, positive Babinski signs	Brain sagging, B/L subdural collection, diffuse dural enhancement	EBP	Headaches resolved	Improved significantly, not mentioned if it fully resolved.	Not done

Weisfelt et. al. 2004	51/M	Yes (6 weeks of headaches)	Yes (1 week cognitive decline and fluctuating consciousness)	Somnolence and apneas	Gait ataxia (details not available)	Fluctuating consciousness level, positive Babinski sign	B/L subdural effusions, pachymeningeal enhancement, descent of cerebellar tonsil, flattening of pons	EBP and lumbar infusion of normal saline	Headache resolved	All neurological symptoms reportedly resolved	Restoration of cerebral descent

Mistry et al.	39/M	Yes (4 months of orthostatic headache, 4 weeks of nausea)	Yes (4 weeks of mood/personality changes)	Diplopia	Gait ataxia (details not available)	No focal neurological signs	Downward displacement of diencephalon, B/L subdural collections	EBP (failed), targeted blood patch (repeated 2 times)	Resolved temporarily on first targeted blood patch before returning	Significantly improved only after 3 targeted blood patches	Not done

Uysal et al. 2008	5/F	Yes (1 month of orthostatic headaches)	no	Sudden onset hearing ess	Gait ataxia (details not available)	Ataxic gait, hearing loss	Enlargement of cervical venous plexus, dural leak at level of L3–L4 vertebraepk	Oral caffeine, EBP × 2	Complete resolution after EBP	Improvement with oral caffeine, Complete resolution with EBP × 2	Not done

Devine et al. 2009	46/M	Yes (headache, neck pain, aural fullness)	Yes (memory disturbance)		Gait unsteadiness	Impaired tandem gait, dysmetria on finger-nose-finger, postural tremor of arms, MMSE 20/30	Brainstem sagging, distortion of midbrain, B/L transtentorial herniation, B/L subdural collections	Bed rest in trendelenburg, IV caffeine, oral dexamethasone	Excellent response	Excellent response	Not done

Sasikumar et al. 2018	64/M	No	no		Broad based stance sway fluctuations, narrow stride length, decrease stride velocity	None other gait abnormality for both cases	Low-lying cerebellar tonsils, diffuse pachymeningeal thickening, subdural effusions	Multiple non-targeted EBP.	Not mentioned	“Remarkable improvements” on quantitative gait analysis 1 week after EBP.	MRI did not show radiologic eveidence of SIH

	80/F	No	No		Details are not mentioned except “side to side” while walking		Venous distention, pachymeningeal thickening and subdural effusions.	Declined treatment		Spontaneous resolution of symptoms	

Domínguez et al. 2023	53/M	Yes (6 months of orthostatic headache and tinnitus)	Yes (2 months of behavioral changes, delusional ideation)		Recurrent falls, gait ataxia with retropulsion	Dysmetria in all limbs, Kinetic tremor of arms, Severe cognitive dysfunction tests	Descent of cerebellar tonsils, transtentorial herniation, distortion of brainstem structures and descent of splenium of corpus callosum, CSF leak at D5 level.	EBP × 2	Sustained recovery of cognitive symptoms	Complete and sustained recovery after EBP (SARA score improved from 16 to 0).	Resolution of brain sagging


**BSS:** Brain sagging syndrome, **CSF:** Cerebrospinal fluid, **MMSE:** Mini Mental Status Examination, **SARA:** Scale for the assessment and rating of ataxia (SARA), **SIH:** Spontaneous intracranial hypotension, **EBP:** Epidural blood patch.

**Table 2:** Summary of publications on SIH/BSS that have reported tremor as a predominant symptom.


AUTHOR, YEAR	AGE/SEX	TYPICAL SIH SYMPTOMS NOTED?	TYPICAL FBSS SYMPTOMS NOTED?	OTHER SYMPTOMS	CHARACTERISTICS OF TREMOR	KEY EXAMINATION FINDING	IMAGING ABNORMALITIES	TREATMENT	IMPROVEMENT OF SIH/FBSS SYMPTOMS?	IMPROVEMENT OF MOVEMENT DISORDERS?	FOLLOW UP BRAIN IMAGING

Turgut et al. 2009	57/M	No	No	none	B/L R > L 7 Hz postural tremor with intention component	Tremor absent on rest, not associated with bradykinesia, rigidity, dystonia.	B/L pachymeningeal enhancement, brain sagging, CSF leak at left thoraco-lumbar area due to a ruptured meningeal diverticula	Epidural blood patch at thoracolumbar junction	–	Complete resolution of tremor at 2 months after EBP	Decrease in meningeal thickening and resolution of brain sagging

Mokri et al. 2014	51/W (patient 2)	Yes (exertional-Valsalva headaches, positional dizziness)	No	Spasmodic torticollis	Dystonic head tremor to the right (side-to-side)	Phasic dystonic head movements to the right with mild dystonic deviation to right.	Pachymeningeal enhancement, descent of cerebellar tonsil, flattening of anterior pons,	Conservative, avoiding provoking factors.	Gradual resolution of headaches at follow up visit in 5 years.	Gradual resolution of dystonia and tremor at follow up visit 5 years later.	None

	52/W (patient 4)	Yes (orthostatic headaches provoked by Valsalva, vertigo, tinnitus)	No	Gait unsteadiness, unspecified	Orthostatic mixed static and movement tremor of upper limbs, more on right.	No exam was mentioned in the article.	Pachymeningeal enhancement, descent of cerebellar tonsil, flattening of anterior pons, obliteration of perichiasmatic cistern, possible CSF leak at S1 level.	EBP every 6 weeks, later IV saline infusions every 3 weeks. S1,S2, and partial L5 hemilaminectomies did not identify definitive leak, area was packed with gelfoam and fibrin glue.	EBP led to transient marked improvement in headaches for 2 weeks.	Complete resolution for 2 weeks after EBP. After surgery, had relief for 6 weeks before return in symptoms.	Improvement but still persistent pachymeningeal enhancement

Salazar et. al. 2016	68/M	Yes (occassional orthostatic headaches)	No	Gait unsteadiness	Bilateral progressive hand tremor over 2 years	Fast, distal kinetic tremor in B/L hands without postural/rest component. Mild dysmetria, truncal titubation, gait start hesitation, broad based gait with impaired tandem gait.	Diffuse infra- and supratentorial pachymeningitis, cerebellar tonsillar descent, mild brain sagging. No leak on CT myelogram.	Conservative (caffeine)	Not mentioned	Caffeine with partial response of symptoms.	Not done.

Iyer et al.	23/M	Yes (orthostatic headaches)	No	Sleepiness in sitting position.	7 month progressive right hand tremor, at rest and when holding objects.	Somnolent, pupillary light-near dissociation and restriction of upgaze. 3–5Hz Holmes tremor in R upper limb	Sagging of brain with transtentorial descent of third ventricle and diencephalon leading to deep brain swelling (more on left brainstem) and obliteration of basal cisterns.	Conservative (hydration, trendelenburg position)	Complete relief of somnolence and headaches.	Improved, with mild persistent kinetic and postural tremor at 1 week, complete resolution at 3 months.	Not done.


**BSS:** Brain sagging syndrome, **CSF:** Cerebrospinal fluid, **SIH:** Spontaneous intracranial hypotension, **EBP:** Epidural Blood patch.

**Table 3:** Case reports on SIH/BSS that have reported parkinsonism as predominant movement disorder.


AUTHOR, YEAR	AGE/SEX	TYPICAL SIH SYMPTOMS NOTED?	TYPICAL FBSS SYMPTOMS NOTED?	OTHER SYMPTOMS	FEATURES OF PARKINSONISM	KEY EXAMINATION FINDINGS	IMAGING ABNORMALITIES	TREATMENT	IMPROVEMENT OF SIH/FBSS SYMPTOMS?	IMPROVEMENT OF MOVEMENT DISORDERS?	FOLLOW UP BRAIN IMAGING

Pakiam et al. 1999	54/W	Yes (headaches worsened on cough, relieved on lying).	Depression that was treated.	Neck stiffness	Soft speech, R hand rest tremor, slowness in ADLs over 1 year, dysphagia	Hypophonia with weak gag, no rigidity, intermittent high frequency tremor, bradykinesia L > R, impaired tandem walk, retropulsion and impaired postural reflexes.	Downward displacement of posterior fossa structures. Elongation of brainstem in AP plane. Dural enhancement in posterior fossa.	EBP	Headaches resolved.	Resolution of symptoms with normal neurological exam by week 5.	Normal position of brainstem and cerebellar tonsils, mild persistence of midbrain elongation.

Mokri et al. 2014	78/F (patient 5)	Yes (no headache, but vertigo and nausea for 2 weeks)	Yes (slowness of thinking, memory difficulty)	–	Rest tremor of upper extremities	Rigidity of upper extremities, short step walking, hyperactive stretch reflexes, difficulty in abstraction and concentration.	Pachymeningeal enhancement, B/L subdural fluid collection. Low lying cerebellar tonsils.	None	At 4 months, cognitive functioned improved to “above average”.	No signs of parkinsonism at 4 moths.	Not done.

Gupta et al. 2021	66/W	No	Yes (personality changes, cognitive decline over 1 year).	–	Right hand tremor, gait slowness	Rest and re-emergent postural tremor of right hand.	Downward displacement of midbrain, cerebellar tonsils, diffuse dural enhancement. CSF Venous fistula at T9–T10 level. Normal PET scan.	Ligation of venous fistula	Reversal of cognition.	Substantial improvement after ligation.	Not available.

Cochran et al. 2021	64/W	No	Possible (drowsiness, depression)	Fatigue	Tremor in arms (R > L) and lips over 1 year, dysarthria	Bradykinesia (R > L), rest and postural tremor of right hand. Nasal speech, mild L facial weakness, dysmetria on finger-to-nose bilaterally.	Crowding of structures in suprasellar cistern, downward shift of optic chiasm, narrowing of 4th ventricle and decent of cerebellar tonsil.	Surgical repair of suspected dural leak at T7 which was repeated at 4 months.	Not mentioned.	After second repair, had sustained improvement of symptoms with no tremor, facial asymmetry, spasm or dysarthria.	Resolution of previous radiologic findings.

Frachet et al. 2023	21/W	Yes (headache, vomiting)	No	Left CN3 palsy, somnolence followed by coma	Right hemi parkinsonism (tremor and rigidity).	Acute stage: Left CN3 palsy, dilated pupils, comatose. After ICU stay: Right sided parkinsonism.	B/L subdural hematoma (L > R), collapse of 3rd ventricle, brain sagging, diffuse pachymeningeal enhancement.	Epidural blood patch	Not mentioned	Gradual resolution of symptoms	Not available.


**AP:** Anterior-posterior, **BSS:** Brain sagging syndrome, **CSF:** Cerebrospinal fluid, **PET:** Positron Emission Tomography, **SIH:** Spontaneous intracranial hypotension, **ICU:** Intensive Care Unit, **EBP:** Epidural Blood patch.

**Table 4:** Case reports on SIH/BSS that have reported chorea.


AUTHOR, YEAR	AGE/SEX	TYPICAL SIH SYMPTOMS NOTED?	TYPICAL FBSS SYMPTOMS NOTED?	OTHER SYMPTOMS	DETAILS OF CHOREA	KEY EXAMINATION FINDINGS	IMAGING ABNORMALITIES	TREATMENT	IMPROVEMENT OF SIH/FBSS SYMPTOMS?	IMPROVEMENT OF MOVEMENT DISORDERS?	FOLLOW UP BRAIN IMAGING

Mokri et al. 2006	59/M	Yes (orthostatic headaches, worsened on coughing, nausea)	Yes (memory complaints, confusion, sleepiness)	Dysarthria, dysphagia	Generalized chorea (face, trunk and extremities)	Worsening of chorea while walking, hyperkinetic dysarthria, and positive Babinski sign	Cerebellar tonsillar descent, T-2 hyperintensity of brainstem, pachymeningeal enhancement, CSF leak in cervico-thoracic area and T8–T9 meningeal diverticula	Epidural blood patch	Resolution of headaches, nausea, speech. Cognitive improvement not mentioned.	Complete resolution at 4 month follow up	None

Mulroy et al. 2017	42/M	Yes (6 months of chronic daily headache)	Yes (behavioral changes- impulsivity, disinhibtion.	Hiccups, Dysarthria, dysphagia	Limb and orofacial chorea and athetosis	Details are not mentioned	Both patients had downward displacement of brainstem and cerebellum. Slight distortion of basal ganglia in patient 1. Probable CSF Leak at T4 in patient 1.	Patient 1: conservatiive treatment, 2 EBP, T4 targeted blood patch	No improvement	No improvement	Not available.

	64/M	Yes (9 months of orthostatic headaches)	No		Limb and orofacial chorea and athetosis	Nasal speech		Patient 2: 2 epidural patches	No improvement	Temporary improvement in chorea but not sustained.	

Fearon et al. 2022	35/M	No	No	Decreased left hand dexterity, falls	Left sided hemichorea	Vertical supranuclear gaze impairment, brisk tendon reflexes, positive Babinski on left	Descent of brainstem, splenium, and cerebellar tonsils along with venous distention. MRI with thoracic epidural fluid collection.	EBP	–	Partial improvement in balance and chorea	Not available

Figueroa et al. 2018	62/M with Huntington’s Disease	Yes (progressive orthostatic headache with nausea and vomiting)	Yes- worsening of progresive cognitive decline and hallucinations	Gait disturbance	Pre-existing facial chorea got generalized	Details are not mentioned, but repeat MoCA with 5 point decrement to 23/30	Pachymeningeal enhancement, sagging of brainstem, subdural hygromas, possible CSF leak at C1–C2	Large volume blood patch (56 ml), and later subdural evacuation	Headache free, resumed work and MoCA back to baseline (29/30) at 3 months	Chorea returned to baseline	Small bilateral subdurals with resolution of brain slumping


**BSS:** Brain sagging syndrome, **CSF:** Cerebrospinal fluid, **EBP:** Epidural blood patch, **SIH:** Spontaneous intracranial hypotension.

## Phenomenology of Movement Disorders in BSS

### Gait unsteadiness

The most common phenomenology reported in BSS is gait unsteadiness. Gait unsteadiness frequently emerges in the backdrop of one or several cardinal symptoms of SIH/BSS. Nowak and colleagues were the first to report paroxysmal ataxia along with visual blurring in a 50-year-old man with CSF leak at T12–L1 level [12]. Patient’s gait improved substantially after EBP. Subsequently, several other publications (Table 1) reported gait unsteadiness as a major presenting symptom along with the classic symptoms of SIH/BSS [13,14,15,16,17]. All these reports are on middle aged patients who improved substantially after EBP or only with conservative therapy [16]. Our literature search revealed only one article that reported ataxia related to SIH/BSS in a child [18]. Uysal and colleagues reported gait ataxia in a five-year-old girl along with chronic headache and hearing loss. Imaging revealed enlargement of cervical venous plexus and a dural leak at L3–L4. Similar to majority of the case reports summarized in Table 1, the child in Uysal et al.’s report improved substantially after EBP [18]. Although gait dysfunction usually co-exists with the classic symptoms/signs of SIH/BSS, rarely, patients may present with isolated gait dysfunction. Sasikumar and colleagues reported isolated gait dysfunction in a 64-year-old man and an 80-year-old woman [19]. In the 64-year-old man, objective gait assessment revealed reduced stride length and velocity along with a broad-based stance and frequent swaying. There were subjective as well as objective improvement in gait after multiple non-targeted blood patch. Gait unsteadiness of the 80-year-old woman improved spontaneously in 6 months [19].

In addition to the aforementioned reports, several case series have reported gait unsteadiness in patients with SIH/BSS. Six out of eight patients in the study by Wicklund et al., 19 out of 29 patients in the study by Schievink et al. and five out of eight patients in the study by Capizzano et al. had gait unsteadiness at presentation [20,21,22]. Radiological evidence of brain sagging was the common finding in all these studies. There were variations in the extent and duration of improvement in the symptoms after EBP [20,21,22].

It is important to note that, due to involvement of the common neuroanatomical substrates, gait dysfunction in SIH/BSS frequently co-exists with other movement disorders. For example, the patient described in the reports by Devin et al., [16] and Domínguez et al., [17] had postural tremor and kinetic tremor of the arms, respectively.

### Tremor

Tremor is the second most commonly reported movement disorder in SIH/BSS (Table 2). It usually co-exists with gait dysfunction of varying severity. In the case series by Schievink et al., 15 out of 29 patients had tremor; however, the exact phenomenology of tremor was not mentioned [21]. In several case reports, the tremor phenomenology was documented as postural and/or kinetic involving both arms [16,17,23,24]. In a case series of five patients, Mokri described two patients with tremor [25]. While one patient had right hand rest and action tremor (without bradykinesia), other had dystonic head tremor (with torticollis). The former experienced partial improvement with EBP and intravenous normal saline infusions. In an interesting report, Iyer and colleagues described a 23-year-old patient with Holmes tremor in the context of SIH/BSS [26]. In addition to Holmes tremor of right hand, the patient had several other clinical signs such as somnolence, upgaze restriction, and pupillary light near dissociation. With conservative measures (increased hydration and rest in Trendelenburg position), the patient had excellent recovery.

In summary, patients with SIH/BSS may present with various tremor phenomenologies (rest, postural, kinetic, dystonic). The most commonly reported phenomenology is postural and kinetic tremor of the arms. Isolated tremor has not been reported as a feature of SIH/BSS. Any patient presenting with subacute onset of tremor in the backdrop of chronic headache, memory issues, or gait unsteadiness should be investigated in detail to explore SIH/BSS.

### Parkinsonism

Several authors have reported subacute parkinsonism in patients with BSS (Table 3). Pakiam and colleagues were the first to publish a report on parkinsonism in a 55-year-old woman with BSS [27]. The patient had asymmetric rest tremor and bradykinesia along with soft speech and gait unsteadiness. The patient had some of the commonly observed radiological signs that include downward displacement of posterior fossa structures and elongation of the brainstem in the anterior-posterior plane. The patient had complete recovery 5-weeks after having EBP. Mokri reported a 78-year-old man with rest tremor along with gait unsteadiness, memory problem, and slowness in thinking [25]. Examination revealed some of the core features of parkinsonism such as rigidity and walking with short steps. The patient had imaging evidence of brain sagging and improved spontaneously in a few months. Parkinsonism may emerge with bulbar symptoms, as reported by Cochran and colleagues in a 64-year-old woman [28]. The patient had parkinsonism in the form of rest and postural tremor of right arm, lip tremor, and bilateral upper extremity bradykinesia along with nasal speech, swallowing difficulty, and dysarthria. Patient was suspected to have a leak at T7 level, and the symptoms improved a few months after the surgical repair. Gupta and colleagues described a patient with parkinsonism in the context of SIH due to a CSF venous fistula at T9–T10 level and the patient improved substantially after ligating of the fistula [29]. Recently, Frachet and colleagues reported post-coma hemiparkinsonism in a patient with SIH/BSS caused by lumbar puncture for intrathecal chemotherapy [30]. The patient had gradual resolution of symptoms after EBP. As SIH usually does not lead to strikingly asymmetric symptoms/signs, it is unclear how the patient presented with hemiparkinsonism. A trial of levodopa, serial imaging of the dopamine transporter and longitudinal clinical evaluation would be valuable in such cases to see if the laterality of symptoms is due to asymmetric mechanical distortion of basal ganglia or due to transient unmasking of underlying dopaminergic deficit.

In the absence of chronic orthostatic or Valsalva headache, or any behavioral symptoms, clinical features mentioned above will be concerning for parkinsonism, either Parkinson’s disease (PD) or atypical forms. Although never reported, a patient with PD can also develop SIH/BSS and manifest with the additional cognitive, behavioral, and gait symptoms associated with the latter. Therefore, subacute worsening of parkinsonism in a patient with PD along with the emergence of new symptoms should not be attributed to natural progression of PD and should warrant detailed investigations to rule out or rule in treatable conditions such as SIH and BSS.

### Chorea

There are a few case reports on chorea in patients with SIH/BSS. Mokri and colleagues reported generalized chorea (face, trunk, extremities) along with the classic symptoms of SIH/BSS in a 59-year-old man who was found to have CSF leak at cervicothoracic level [31]. Chorea used to be pronounced while walking. The patient recovered completely 4 months after having EBP. Upper limb chorea was reported in studies by Wicklund et al. and Capizzano et al., [20,22]. Mulroy and colleagues reported chorea-athetosis in two cases of SIH/BSS [32]. These authors documented orofacial, lingual, and limb choreoathetosis in both cases. While epidural blood patch did not result in any improvement one patient, other had partial improvement for a short period. Although extremely rare, hemichorea may be a presenting sign of SIH/BSS [33]. However, similar to the patient with hemiparkinsonism mentioned above, it is unclear how SIH/BSS leads to asymmetric chorea and more studies are needed to obtain better insights.

SIH/BSS patients with chorea, who also have memory disturbance and several behavioral symptoms such as apathy, impulsivity, and disinhibition, can be easily mistaken for Huntington’s disease (HD), although the lack of positive family history should raise the suspicion of a different condition. BSS can be differentiated from HD also by the presence of postural/exertional headache. As described in the context of PD above, rapid worsening of chorea or neuropsychiatric symptoms in HD should not be attributed to the natural progression of HD and secondary etiologies should always be explored. For instance, Figueroa and colleagues reported a case of HD wherein rapid clinical worsening was found out to be secondary to BSS (due to CSF leak at C1–C2 level) [34]. The patient returned to baseline after epidural blood patch. In fact, patients with neurological conditions are often prone to falling and related trauma can contribute to CSF leakage.

### Compulsive repetitive flexion with breath holding

In a recently published study, Schmahmann and Scheivink reported a specific sign that could be pathognomonic of BSS [35]. The study involved 51 patients with clinical and radiological features of BSS and reported repetitive flexion with breath-holding in 13 patients at presentation, and in 5 patients in whom these specific symptoms resolved prior to presentation. These repetitive movements were intermittent truncal and hip flexion, with a frequency of once in every 1–2 minutes and each episode lasted for 15–20 seconds. The authors hypothesized that those movements probably conferred some clinical benefit by decreasing the CSF-to-venous pressure gradient with breath holding (which would increase venous pressure), hence minimizing the loss of CSF from the arachnoid system. While the exact phenomenology has not been mentioned in other reports, the tendency to lean forward with hip flexion has been described [21,36]. In the case report by Park and Kim, there was an acute onset truncal flexion, diagnosed as “camptocormia”, in a patient with SIH [36]. As the truncal flexion is presumed to be a compensatory strategy to reduce severe orthostatic headache, it is likely that the pathophysiology of this postural abnormality is different than what is seen in PD, multiple system atrophy or axial myopathies [37,38,39].

### Other movement disorders

Another hyperkinetic phenomenology reported in BSS is orofacial dyskinesia, which was present in 6 out 29 patients reported by Schievink et al., [21]. The authors noted that the orofacial dyskinesia was associated with downward displacement of the corners of the mouth in 5 patients. A case of hemifacial spasm, in conjunction with headaches, was also reported by Chow et al., which subsequently improved on repeat epidural blood patches (and thereafter resolved on treatment with botulinum toxin) [40]. As mentioned above, in the case series by Mokri, one patient presented with spasmodic torticollis to the right (and side-to-side head tremor) that began after 5 years of exertional-Valsalva headaches with only partial benefit to 3 cervical EBPs [25].

## Pathologic Basis of the Emergence of Movement Disorders in SIH/BSS

CSF in the skull not only serves as a mechanical buffer to protect the brain, but also provides buoyancy, effectively reducing the net weight of the brain suspended in the cranial cavity. In spontaneous intracranial hypotension, there is a depletion of CSF volume, thus reducing the buoyancy effect of CSF. As a result, there is a gravitational descent of the brain within the skull. In terms of the clinical symptoms, orthostatic headache is characteristic of SIH, and is due to the traction exerted by the brain’s downward displacement on meningeal pain fibers. In BSS, the downward displacement of the brain may exert mechanical stress on frontotemporal cortical structures or their connecting circuits, potentially disrupting normal cognitive and behavioral functions [20].

**Figure 1:**
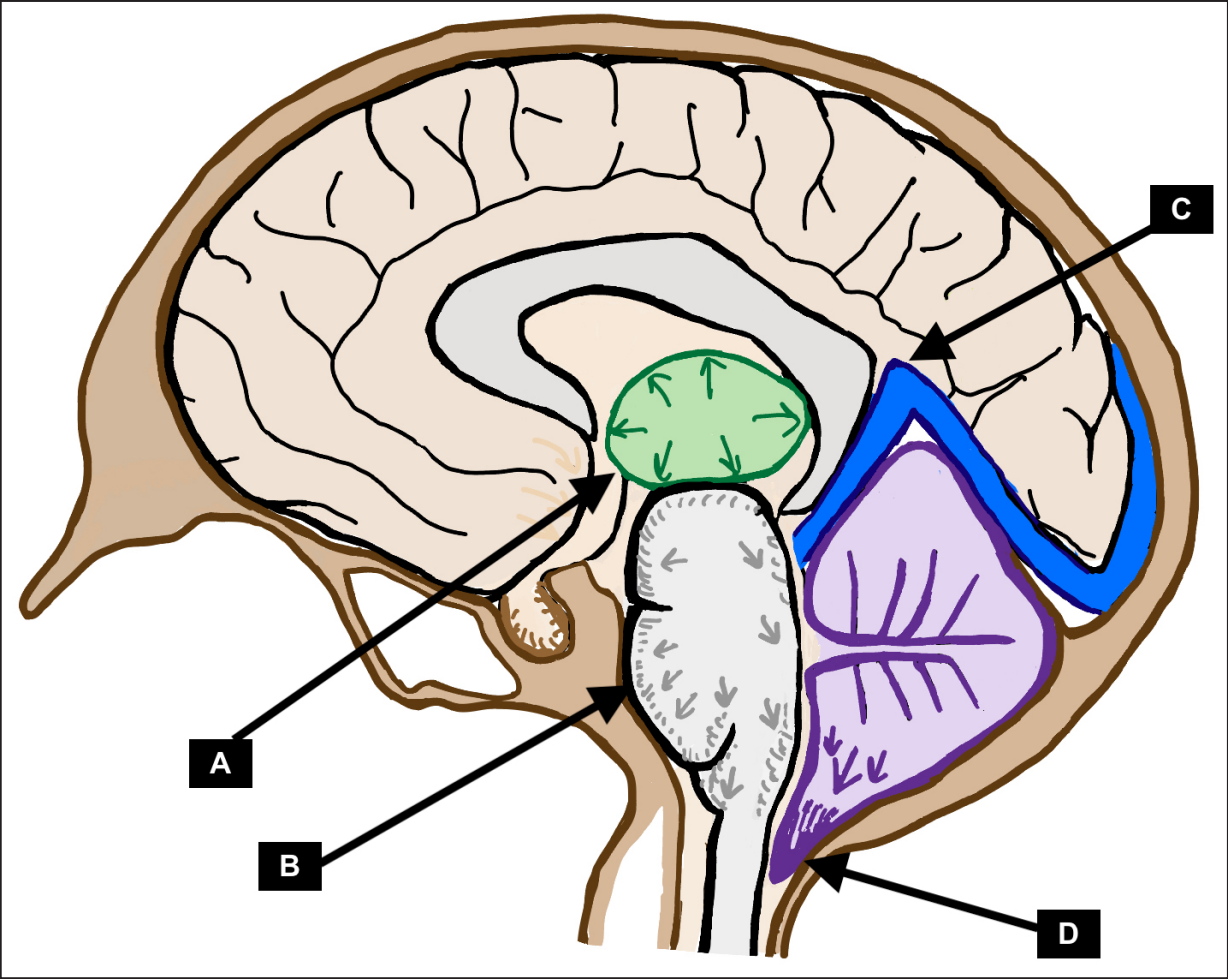
Pathophysiology of movement disorders in brain sagging syndrome due to spontaneous intracranial hypotension: **A.** Vasogenic edema in subcortical structures such as the thalamus or putamen. **B.** Edema of the brainstem from venous stagnation. **C.** Reduction in angle between the vein of Galen and the straight sinus, creating a functional stenosis that worsens venous stagnation. **D.** Cerebellar tonsillar ectopy due to deep brain edema and cerebellar hypoperfusion (from venous hypertension) causing cerebellar symptoms.

In terms of movement disorders, a variety of related pathophysiologies can be postulated. Due to a decrease in CSF volume, there is a compensatory venous engorgement (as per the Monro-Kellie doctrine) that can lead to venous stagnation, subsequent venous hypertension, finally leading to vasogenic edema particularly in the deep brain structures such as the putamen or thalamus [41]. Furthermore, this venous stagnation is particularly prominent at the confluence of the vein of Galen into the straight sinus, and due to the above-mentioned vasogenic edema, this can reduce the angle between the vein of Galen and the straight sinus, thereby causing a functional stenosis that will further worsen venous stagnation and perpetuate a vicious cycle of worsening deep brain vasogenic edema [42]. In addition, direct mechanical stress on subcortical structures such as putamen, as well as downward herniation of cerebellar tonsils (due to the deep brain edema) may also be one potential cause for symptoms. Finally, venous hypertension can also reduce the perfusion in affected areas, particularly in the cerebellar regions [43]. Figure 1 highlights the key pathophysiologic elements related to BSS.

## Diagnosis and Management

SIH/BSS should be included in the list of differential diagnoses in any patient who presents with the above-mentioned involuntary movements in the backdrop of orthostatic headaches, especially when accompanied by subacute behavioral/cognitive symptoms. Brain MRI with and without contrast is the gold standard to confirm brain sagging. The salient imaging findings of the various cases presenting with movement disorders are detailed in Tables 1, 2, 3, 4. The most commonly reported findings are sagging of the brainstem towards the clival bone, downward cerebellar tonsillar ectopy, bitemporal or transtentorial herniation. Signs of intracranial hypotension, such as bilateral subdural effusions and pachymeningeal enhancement, are also common. There are several studies that reported venous abnormalities, such as dural venous engorgement, cervical venous plexus dilation, and reduction of the angle between the vein of Galen and the straight sinus in a midsagittal cut. A definite CSF leak may not be found in half of the cases [44,45].

Treatment-wise, conservative approaches like increased fluid intake, bed rest/maintenance of Trendelenburg position, consuming caffeine, theophylline, and analgesics, appear to be of limited utility, but may be tried [7]. The primary treatment recommended for BSS is administering a lumbar EBP [2]. This can cause considerable improvement in symptoms, though in many cases, relief can be transient. Should the exact location of a CSF leak be identified, a targeted blood patch could serve as a more specific treatment [2]. Surgical correction for structural defects or pinpointed CSF leaks may be warranted depending on the source of the CSF leak and the extent of the dural defect [46]. Blood patches have not been effective for treating CSF-venous fistulas, which necessitate surgical ligation. In some cases, a steroid regimen has proven effective for BSS and may be considered if EBP fails [47,48]. Additionally, the administration of intrathecal saline has been explored and can offer short-lived benefits [49,50]. A detailed discussion of the management of BSS and SIH is beyond the scope of this review and we refer the readers to several excellent review articles on this topic [1,3,5]. In the case reports mentioned in this review, a considerable proportion of patients did not have complete resolution of movement disorders despite above treatment. However, many of these case reports did not have follow-up imaging to review if the underlying pathophysiology (i.e. brain sagging) was fully corrected, and definitive treatment (surgical correction) was not performed. if comprehensive treatment approaches fail to resolve movement disorders even after correcting the sagging due to hypotension, the connection between brain sagging and movement disorders may need to be reconsidered in such cases.

## Conclusion

BSS is a potentially reversible condition typically characterized by chronic orthostatic headache and behavioral/cognitive changes. Ten percent of patients with BSS may manifest various involuntary movements. The underlying pathophysiology likely involves venous engorgement of deep brain structures and the brainstem, direct mechanical stress on subcortical structures, and tonsillar ectopy. Given that it is treatable with blood patches, subacute onset of movement disorders in the context of chronic headache or memory disturbance, should prompt inclusion of BSS in the list of differential diagnoses. Therefore, movement disorders neurologists must be acquainted with BSS and its manifestations.
